# Serum vitamin D levels of patients with oral squamous cell carcinoma (OSCC) and expression of vitamin D receptor in oral precancerous lesions and OSCC

**DOI:** 10.4317/medoral.20368

**Published:** 2015-02-07

**Authors:** Martin Grimm, Marcel Cetindis, Thorsten Biegner, Max Lehman, Adelheid Munz, Peter Teriete, Siegmar Reinert

**Affiliations:** 1 Department of Oral and Maxillofacial Surgery, University Hospital Tuebingen, Osianderstrasse 2-8, 72076 Tuebingen, Germany; 2 Department of Pathology, University Hospital Tuebingen, Liebermeisterstrasse 8, 72076 Tuebingen, Germany; 3 Cancer Research Center, Sanford-Burnham Medical Research Institute, 10901 North Torrey Pines Road, La Jolla, CA 92037, USA

## Abstract

Background: Resistance to programmed cell death (apoptosis) is a crucial factor for the carcinogenesis of oral squamous cell carcinoma (OSCC). Vitamin D (calcitriol) may overcome apoptosis resistance in tumor cells of OSCC. Vitamin D receptor (VDR) expression in oral precancerous lesions of OSCC has not been analyzed and serum vitamin D level seems to be a predictor of cancer development. Material and Methods: Expression of VDR was analyzed in normal oral mucosa (n=5), oral precursor lesions (simple hyperplasia, n=11; squamous intraepithelial neoplasia, SIN I-III, n=35), and OSCC specimen (n=42) by immunohistochemistry (IHC). Moreover, serum vitamin D levels were measured by 25(OH)D3 (calcidiol) in patients with OSCC (n=42) and correlated with IHC results. Results: Expression of VDR was significantly increased in precancerous and OSCC compared with normal tissue. Compared with SIN I-III lesions VDR expression significantly decreased in OSCC. Severe vitamin D deficiency was detected in our OSCC patient cohort but there was no significant correlation analyzed between serum vitamin D levels and corresponding immunohistochemically detected VDR expression in OSCC. Conclusions: Our survey provides the first evidence of VDR expression in precancerous lesions of OSCC. Apoptosis induction of VDR+ cells in oral precancerous lesions and OSCC by natural vitamin D or synthetic vitamin D compounds could be useful for chemoprevention. Moreover, systemically and/or locally applied, these compounds may act as sensitizers for apoptosis mediated by radio-, and chemotherapy treatment in OSCC.

** Key words:**Oral cancer, oral precancer, lichen planus, leukoplakia, apoptosis, serum 25(OH)D3, vitamin D receptor, chemoprevention, multistep carcinogenesis.

## Introduction

The development of oral squamous cell carcinoma (OSCC) is a multistep process that influence key cellular pathways involved in tumor development and growth. A diversity of endogenous and exogenous stimuli is leading to a complex series of molecular changes participating in cancer development (1,2). The subsequent rise of malignant tumors from a single transformed cell and the development through detectable pre cancerous stages is described by the model of multistep carcinogenesis (3). This fact depicts the need for continued efforts to discover new biomarkers for the early OSCC diagnosis and therapy as well as for the improved understanding of disease pathogenesis as a first step towards improving treatment. The pace of clinical translation in the field of molecular translational oncology has accelerated in the last few years. Analyzing the mechanistic basis of the multistep process potentially allows the development of molecular tools to manipulate oral carcinogenesis, which is relevant to the practicing oncologist.

OSCC is a major cause of morbidity and mortality worldwide with low response to chemotherapy and basic resistance to most standard anticancer drugs (4,5). The process of programmed cell death (apoptosis) that may occur in multi cellular organisms is a genetically regulated cell death involved in the deletion of cells in normal as well as malignant tissues (6). Resistance to apoptosis (7) is known as a crucial factor for the carcinogenesis of OSCC, which is associated with tumor recurrence as well as radio-, and chemotherapy resistance (8,9). Therefore, overcoming apoptosis resistance is a major aim in the treatment of OSCC.

In a number of cell types, the antineoplastic activity of 1,25-dihydroxyvitamin D3 (1,25-(OH)2D3, calcitriol) contributes to the induction of apoptosis, inhibition of invasiveness, and angiogenesis (10,11). The antineoplastic activity of 1,25-(OH)2D3 was shown in-vitro and in-vivo in a wide variety of malignancies including head and neck cancer (12-18) and specifically OSCC (19-21). Moreover, the effectiveness of cytostatic chemotherapy to induce apoptosis in OSCC cells is enhanced by calcitriol (21).

In general, the measurement of serum calcidiol is a better predictor of cancer development than calcitriol (1,25 (OH)2D3) (22,23). Studying serum vitamin D level and its corresponding vitamin D receptor (VDR) seems to be reasonable for guiding supportive treatment of patients with pre cancerous lesions and OSCC.

Recently, we have demonstrated low VDR expression as an adverse prognostic factor for the survival of patients with OSCC (11). However, the expression of VDR in different pre cancerous lesions has not been described yet. Therefore, the purpose of this study was to investigate VDR expression in pre cancerous lesions. Moreover, serum vitamin D levels (calcidiol) were measured and correlated with tissue-related VDR expression in OSCC patients.

## Material and Methods

- Patients and Tumor Specimen 

The records of healthy individuals (normal oral mucosal tissues, n=5), patients with oral precursor lesions (simple hyperplasia, n=11; squamous intraepithelial neoplasia SIN I, n=5; SIN II, n=9; SIN III, severe dysplasia, n=10; SIN III, carcinoma in situ, n=11), and patients with invasive OSCC (n=42,  Table 1) were retrospectively assessed from January 2009 to December 2013. Out of these 42 precursor lesions, 4 specimens were clinically and histopathologically classified as oral lichen planus and 38 specimens presented as leukoplakia. The diagnosis of normal oral mucosal tissues, precursor lesions, and invasive squamous cell carcinoma was confirmed by the department of Pathology, University Hospital Tuebingen. The material was archival formalin-fixed, paraffin-embedded tissue from routine histopathological work-ups. The material has been stored with permission of the local ethics committee of the University Hospital Tuebingen (approval number: 562-2013BO2), after informed consent obtained from the patients prior to surgical resection. Tumor blocks of paraffin-embedded tissue were selected by experienced pathologists, evaluating the routine H&E stained sections. Sections from all available tissues underwent histopathological assessment, blinded to the prior histopathology report. Serial tissue sections (2 µm thickness) were cut from formalin-fixed paraffin-embedded (FFPE) blocks on a microtome and mounted from warm water onto adhesive microscope slides. First, we assessed H&E sections from each tissue section to differentiate between normal tissue, precursor lesions, tumor cell areas, stromal areas, and infiltrating immune cells. Oral precursor lesions were classified according to WHO criteria (3). Tumor staging was performed according to the 7th edition of the TNM staging system by the UICC/ AJCC of 2010. Grading of OSCC was defined according to WHO criteria.

**Table 1:**
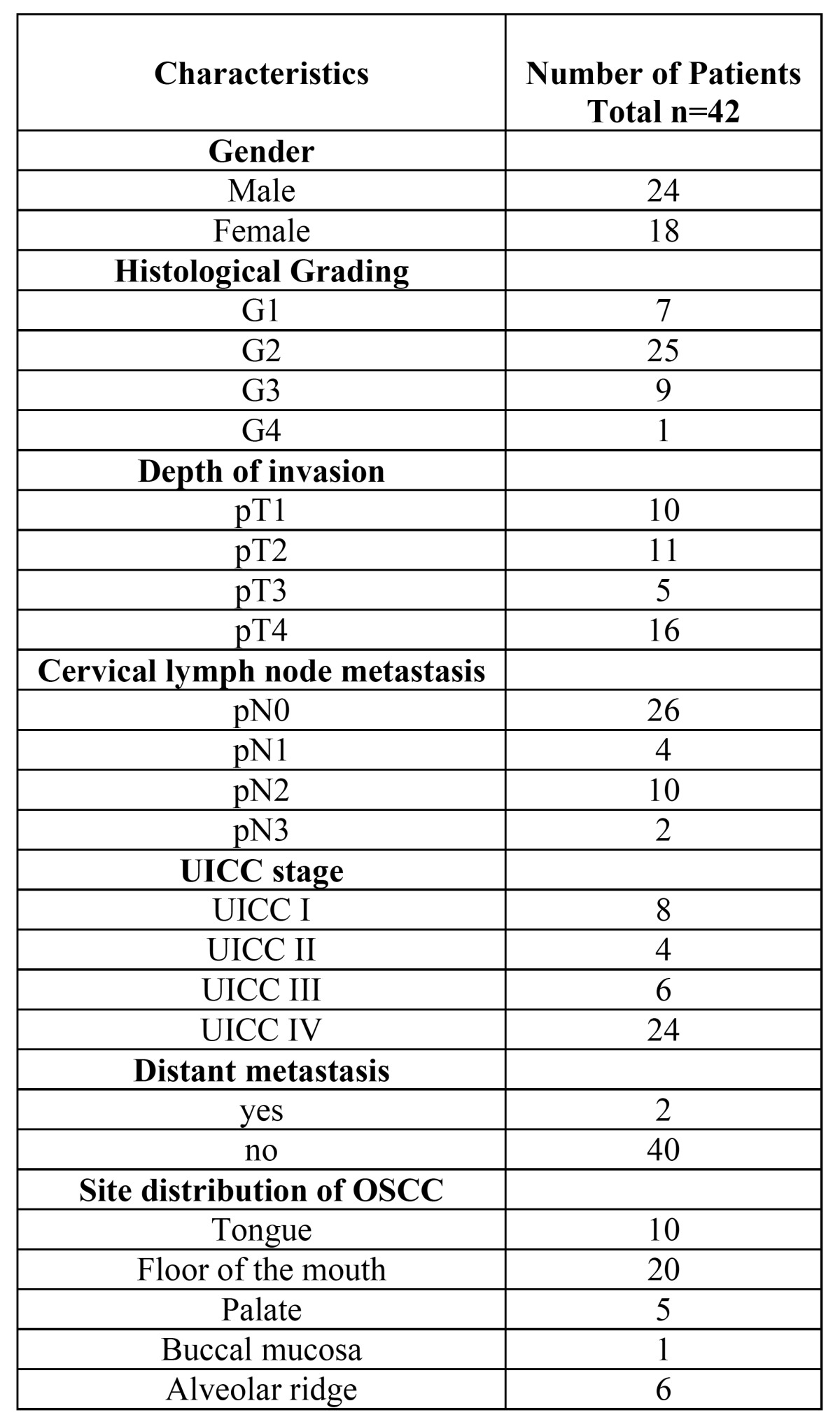
Clinicopathological characteristics of 42 patients with OSCC.

- Staining procedure and quantification of immunohistochemistry

We stained for VDR (LifeSpan Biosciences, Eching, Germany, rabbit anti-human VDR pAb, LS-B2976, dilution: 1:100, (11) in tissue sections. Staining was performed on serial sections of 2µm thickness, which were deparaffinized in xylene and ethanol and rehydrated in water. Heat induced epitope retrieval (HIER) was performed with citrate buffer pH 6.0 (Dako, Hamburg, Germany). Endogenous peroxidase activity was quenched with 0.3% hydrogen peroxide. Endogenous biotin activity was blocked using the avidin/biotin blocking kit (Vector Laboratories, Burlingame, CA, USA). After incubation with the primary or control antibody the Dako LSAB2 peroxidase System (Dako, Hamburg) was used. Slides were subsequently incubated for 5 minutes in DAB (3,3’-diaminobenzidine, Biogenex) counterstained with haemalaun and mounted with Glycergel (Dako).

Five representative high power fields (1 HPF = 0.237 mm2, original magnification: x200-fold) were analyzed for VDR expression in normal tissue, oral precursor lesions, tumor tissue and averaged, respectively. The extent of the staining, defined as the percentage of positive staining areas of tumor cells in relation to the whole tissue area, was semi-quantitatively scored on a scale of 0 to 3 as the following: 0, <10%; 1, 10–30%; 2, 30-60%; 3, >60%. The intensities of the signals were scored as 1+, 2+, and 3+. Then, a combined score (0–9) for each specimen was calculated by multiplying the values of these two categories (24). Cases were classified as negative, 0 points, positive, 1-9 points. Two observers blinded to the diagnosis performed scoring on identical sections marked by circling with a water-resistant pencil and finally with diamond-tipped pencil on the opposite side of the microscopic slide. Pictures were analyzed using a Canon camera (Krefeld, Germany). The photographed images were imported into the Microsoft Office Picture Manager.

- Measurement of serum 25(OH)D3 (Calcidiol) concentrations in patients with OSCC

From patients with invasive OSCC (n=42), in each case, serum samples were obtained in the preoperative period and stored at -80°C until 25(OH)D3 was measured. Serum 25(OH)D3 levels were measured twice by radioimmunoassay at Biovis laboratory (Limburg-Offheim, Germany). History of cancer patients gave no indication for severe kidney or hepatic dysfunction. Serum levels >35 ng/ml, 25-35 ng/ml, 12.5-25 ng/ml and <12.5 ng/ml were considered as normal, mild, moderate and severe vitamin D deficiency, respectively (25).

- Statistical analysis

Statistical analysis was performed with MedCalc Software, Version 13.1.1 (Mariakerke, Belgium). Data were analyzed using the non-parametric Mann-Whitney U Test or Kruskal-Wallis test when more than 2 groups were compared. Correlation analysis of immunohistochemically detected VDR expression with serum 25(OH)D3 was performed by the non-parametric Kendall’s tau (τ) coefficient. All p-values presented were 2-sided and *p* < 0.05 was considered statistically significant.

## Results

- VDR expression in normal mucosa, oral precursor lesions and OSCC

**Figure 1:**
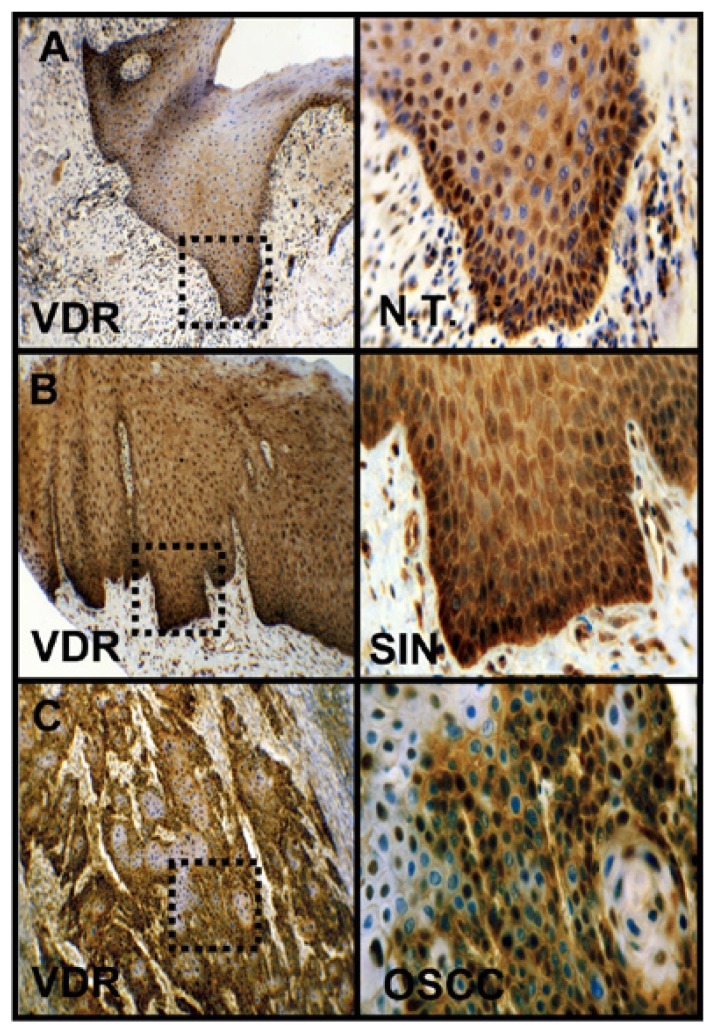
Immunohistochemical staining of VDR in OSCC. Immunohistochemical staining shows representative images of VDR in N.T. (A), SIN (B), and OSCC (C). Brown chromogen color (3,3'-Diaminobenzidine) indicates positive staining, the blue color shows the nuclear counterstaining by hematoxylin. The square box demonstrates the area of interest (original magnification: x100-fold, left panel) which is also shown in larger magnification (x200-fold, right panel). VDR, vitamin D receptor; SIN, squamous intraepithelial neoplasia; N.T., normal tissue.

**Figure 2:**
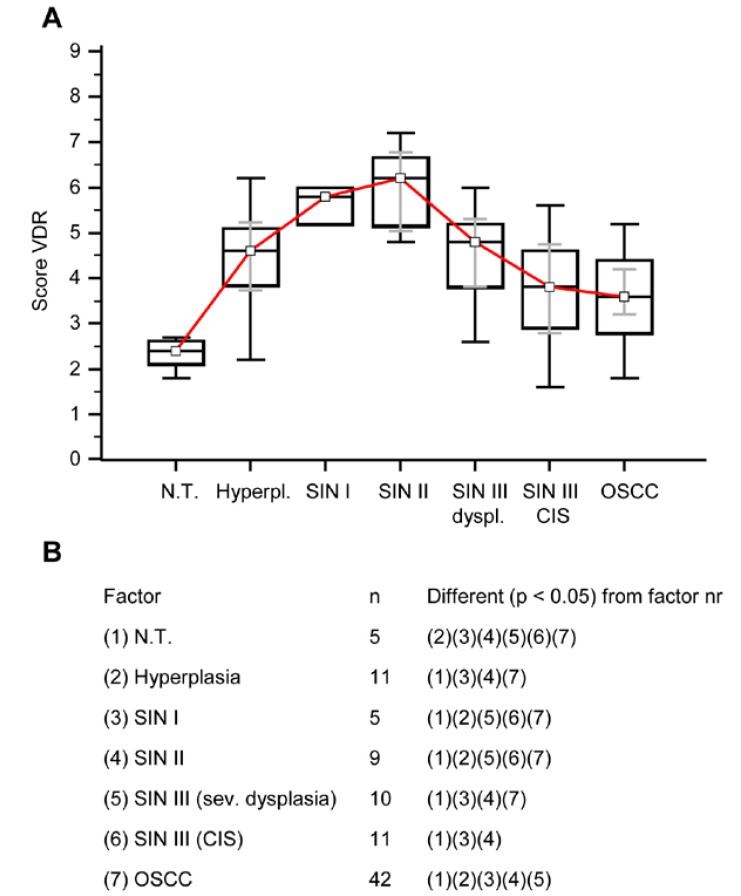
Immunohistochemical analysis of VDR in normal oral mucosal tissue, oral precursor lesions - hyperplasia, SIN, and invasive OSCC. In comparison with normal tissue a significantly increased expression of VDR is observed in hyperplasia, SIN I - SIN III lesions, and OSCC (*p* < 0.05, Kruskal-Wallis Test; A and B). In comparison with SIN I, SIN II, and SIN III (sev. dysplasia) a significantly decreased expression of VDR is observed in OSCC. VDR expression is significantly decreased in OSCC compared with SIN I-III (*p* < 0.0001, Mann-Whitney U Test). Analysis refers to averaged scores. Red line indicates VDR expression results in carcinogenesis. Grey lines show 95% confidence intervals. Analysis of significant statistically different single values is indicated in the table below (B). SIN III is subdivided in severe dysplasia and carcinoma in situ (CIS). VDR, vitamin D receptor; SIN, squamous intraepithelial neoplasia; N.T., normal tissue.

VDR was found expressed in all tissue types (Fig. 1 and 2), normal oral mucosa (n=5/5), oral precursor lesions (simple hyperplasia, n=11/11; squamous intraepithelial neoplasia, SIN I-III, n=35/35), and OSCC specimen (n=42/42). In comparison to normal tissue a significantly (*p* < 0.05) increased expression of VDR (Fig. 1 and 2) was observed in tumor cells of OSCC. Compared with SIN I-III VDR expression was significantly decreased in OSCC (Fig. 1 and 2).

- Serum 25(OH)D3 (calcidiol) values in patients with OSCC and correlation with corresponding immunohistochemically detected VDR expression 

No patient (n=0/42, 0%) showed normal (>35 ng/ml) serum vitamin D levels or mild (25-35 ng/ml) vitamin D deficiency. In 16 out of 42 patients (n=16/42, 38%) moderate (12.5-25 ng/ml) vitamin D deficiency was analyzed and in 26 out of 42 patients (n=26/42, 62%) severe (<12.5 ng/ml) vitamin D deficiency was detected. The mean serum vitamin D level was measured at 12.2 ng/ml (range: 4.0-19.9 ng/ml) in all OSCC patients.

There was no significant correlation found between serum 25(OH)D3 values and corresponding immunohistochemically detected VDR expression in patients with OSCC (n=42): т = 0.114, *p* = 0.2916.

## Discussion

In our survey, we analyzed for the first time VDR expression in different precancerous lesions of OSCC. In patients with OSCC corresponding vitamin D levels were investigated and correlated with immunohistochemical VDR expression. We analyzed increased expression of VDR in OSCC compared with normal oral mucosa. However, compared with SIN I-III VDR expression was significantly decreased in OSCC.

To explain tumor heterogeneity and inherent differences of tumor-regenerating capacity two cancer models are described (26). The clonal selection model of multistep carcinogenesis describes that a random solitary cell undergoes malignant transformation, accumulates multiple mutations and subsequently acquires a survival advantage, which leads to clonal selection (27,28). In contrast, the cancer stem cell (CSC) hypothesis hypothesis regards malignant transformation as a process, occurring in a subset of normal stem cells with pluripotent properties, which underlie deregulation of self-renewal pathways (29,30). Although VDR expression has not been described as a classical CSC marker, putative CSC of the breast (31,32) and OSCC (11) express this receptor. CSCs themselves may undergo clonal evolution and therefore, both models are not mutually exclusive (33,34). Our results of decreased VDR expression in OSCC compared with SIN lesions can be explained with the clonal selection model of carcinogenesis, which proposes that there is a subsequent clonal selection of cancer cells or putative CSC during carcinogenesis (26,32,35). These CSC are resistant to standard adjuvant therapeutic approaches. Enhancing apoptosis in precursor and tumor cells is a key event for cancer therapy and chemoprevention (36). In this connection, natural vitamin D or synthetic vitamin D compounds (10,37) have been demonstrated for induction of apoptosis and play an emerging role in cancer therapy. Several studies have suggested a link between low serum vitamin D levels and an increased risk of cancer (38,39), overall with the strongest evidence for breast and colorectal cancer. Moreover, higher vitamin D intake is linked with a lower risk for breast cancer (40). Recent data indicate that the safe upper intake level for Calcitriol is 10,000 IU/d (41), which is substantially more potent than ergocalciferol (vitamin D2). Authors assume that induction of apoptosis in VDR+ precancerous lesions (e.g. leukoplakia or oral lichen planus) and tumors by vitamin D could be useful for chemoprevention or may act as sensitizers for apoptosis in the treatment of OSCC (37). This hypothesis has to be evaluated in clinical studies. In a Murine Model of Cutaneous Squamous Cell Carcinoma oral calcitriol supplementation enhanced Photodynamic therapy-induced tumor cell death (42). Therefore, topical and/or systemically applicated vitamin d (or in combination with vitamin A derivates (21) may be considered as a possible new, nontoxic, adjuvant cancer therapy, which can be easily introduced into the classic protocols of clinical cancer therapy without any supplementary risk for VDR+ patients. With specific regard to the hypothesis of the field cancerization (43,44) systemically applicated vitamin d seems very meaningful as the tissue of the gastrointestinaltract is exposed to the two main exogenous carcinogenic factors, tobacco and alcohol (45). If the carcinogenic stimuli continue, second metachronous tumors in related sites may occur (45).

From our results, we don´t know whether increased VDR expression in oral pre cancerous or OSCCs exhibits a functionally inactivating mutation and this may be due to a feedback loop coupled with an expression by the tumour cells of a defective VDR (46). In different cell types, Calcitiol has been shown to enhance VDR expression on gene and protein level in vitro (47,48). It has been assumed that the increase of VDR expression following calcitriol administration is due to an increased receptor protein lifetime (48) and/or elevated transcription of VDR genes (49) The study by Lehmann *et al*. indicated that human keratinocytes may have the capacity to hydroxylate vitamin D at the C-1 and C-25 positions (50). Keratinocytes could be able to synthesize the biologically active calcitriol, therefore. However, we don´t know if oral precancerous or OSCCs are able to synthesize calcitriol as well. It can be speculated whether the increased content of VDR on the mRNA and protein levels that we have shown in this study is due to an increased formation of calcitriol in these tumour cells. Moreover, inflammatory peptides/cytokines expressed by tumour-infiltrating leucocytes and oral precancerous/tumor cells may upregulate VDR expression in adjacent cells (51).

Overall, the vitamin D status may be associated with disease-free survival and overall survival time in patients with squamous cell carcinoma of the upper aerodigestive tract (52). Evidence from the literature suggests the relationship between serum calcidiol concentration and cancer prevention as an optimal level of 80 nmol/L (~32 ng/ml) (22,41). Based on the literature review on vitamin D status in the Central Europe (CE) populations, it can be concluded that 25-OH vitamin D levels are on average below the 30 ng/mL level. In wintertime 25-OH vitamin D values of the CE population are close to 21-23 ng/mL for all studied age groups, with a significant increase of 25-OH vitamin D in August reaching 42 ng/mL for those aged 0-9 years, but only 21 ng/mL for the elderly aged 80-89 years (53). Focusing on head and neck squamous cell carcinoma (HNSCC), a previous study by Arem *et al*. (54) anaylzed a median serum 25-OH vitamin D level at 31 nmol/L (~12 ng/ml) without association of oropharynx cancer in Finnish men (including n=131 patients with OSCC). Our results are well in line with the results by Arem *et al*. (54) demonstrating a severe vitamin D deficiency in our patient cohort (mean serum vitamin D level 12.2 ng/ml), which is lower compared with `normal´ CE population as described by Pludowski *et al* above (53).

However, a previous published study by Yuan *et al*. (55) strongly supports a crucial role for vitamin D signaling in oral keratinocyte pathophysiology in-vitro and in-vivo but vitamin D deficiency alone seems to be insufficient to alter oral epithelial homeostasis and provoke carcinogenesis. Afzal *et al*. (56) demonstrated a low plasma 25-hydroxyvitamin D level with increased risk of tobacco-related cancer including HNSCC. In this context vitamin D may conversely modify the carcinogenicity of tobacco smoke chemicals. Authors hypothesize that especially smokers my benefit from Vitamin D intake as more than 80% of OSCC are associated with tobacco abuse.

Oral keratinocytes are able to synthesize the biologically active calcitriol. The steroid hormone responsiveness is directly proportional to the number of corresponding receptors (47). Therefore, we perfomed correlation analysis of serum vitamin d levels and the corresponding VDR in the tumor tissue of OSCC patients but the data did not show any significant correlation. This can be explained as many cytokines and inflammatory peptides excreted by tumour-infiltrating leucocytes as well as tumour cells may influence VDR expression (51).

As suggested for multistep carcinogenesis (2) it is unclear based on the actual literature whether vitamin d can be standardized effective for chemoprevention in the treatment of precursor lesions or OSCC development but it provides a clear rational for further studies in the carcinogenesis of OSCC.

## Conclusions

Our survey provides the first evidence of VDR expression in of OSCC. Apoptosis induction of VDR+ cells in oral pre cancerous lesions and OSCC by natural vitamin D or synthetic vitamin D compounds (10) could be useful for chemoprevention. Moreover, systemically and/or locally applied, these compounds may act as sensitizers for apoptosis mediated by radio-, and chemotherapy treatment in OSCC.
